# Nomograms Combining Three Different Lymph Node Classifications to Predict the Survival of Tonsillar Squamous Cell Carcinoma Patients Undergoing Surgical Treatment

**DOI:** 10.7150/jca.98658

**Published:** 2025-07-28

**Authors:** Dilong Yu, Zhuo Tan, Chuanming Zheng, Jiajie Xu, Ping Huang, Shiqin Hong, Qing Li, Yiwen Zhang, Minghua Ge

**Affiliations:** 1 Jinzhou Medical University, Department of postgraduate education, Jinzhou, Liaoning Province, China.; 2 Otolaryngology & Head and Neck Center, Cancer Center, Department of Head and Neck Surgery, Zhejiang Provincial People' s Hospital (Affiliated People's Hospital), Hangzhou Medical College, Hangzhou 310014, Zhejiang, China.; 3 Zhejiang Provincial Clinical Research Center for Head & Neck Cancer, Hangzhou 310014, China.; 4 Zhejiang Key Laboratory of Precision Medicine Research on Head & Neck Cancer, Hangzhou 310014, China.; 5 Clinical Pharmacy Center, Department of Pharmacy, Zhejiang Provincial People's Hospital, Affiliated People's Hospital, Hangzhou Medical College, Hangzhou, Zhejiang, China.

## Abstract

***Background*:** Tonsillar squamous cell carcinoma (TSCC) is characterized by a high tendency to metastasize to lymph nodes, significantly impacting the treatment modality and recurrence rates in head and neck cancer patients. Therefore, the development of accurate predictive models, such as nomograms, is imperative for the early identification of risk factors associated with lymph node involvement. Various lymph node classification systems, including the number of positive lymph nodes (NPLNs), the ratio of positive lymph nodes (pLNRs), and the logarithm of the odds of positive lymph nodes (LODDS), have been proposed to provide prognostic information. However, the optimal system for classifying lymph nodes remains uncertain, necessitating further investigation to determine which system offers the most accurate prediction of patient outcomes. Thus, our objective was to identify the most effective prognostic nomogram for predicting outcomes in TSCC patients.

***Material and Methods:*** In this study, we retrospectively analyzed data from 1,775 TSCC patients extracted from the Surveillance, Epidemiology, and End Results (SEER) database, following predefined criteria for inclusion. We evaluated the performance of prognostic models using Harrell's concordance index (C-index) and Akaike information criterion (AIC). Subsequently, variables were utilized to construct nomograms for predicting cancer-specific survival and overall survival. Nomograms' predictive capabilities were assessed using Integrated Discrimination Improvement (IDI) and Net Reclassification Improvement (NRI).

***Results:*** The nomogram comprising pLNR, LODDS, and NPLN showed superior efficacy in predicting the survival outcome of patients with laryngectomy for TSCC.

***Conclusion:*** The nomograms developed in this study have the potential to serve as valuable tools for forecasting patient survival following surgical interventions for TSCC.

## Introduction

Cancers affecting the larynx, oropharynx, and oral cavity fall under the category of head and neck cancers. Among these, squamous cell carcinoma (SCC), including tonsillar SCC (TSCC), is notably prevalent and frequently encountered within head and neck squamous cell carcinoma cases^1^, ^2^. Notably, the majority of tonsil carcinomas are attributed to human papillomavirus infection^3^. Recent advancements in the therapeutic landscape have expanded treatment options for early-stage TSCC, predominantly emphasizing surgical interventions and radiotherapy^4^. However, despite these advancements, the prognosis for TSCC remains uncertain and unfavorable, with a considerable proportion of individuals experiencing recurrence within 20 years post-diagnosis, leading to suboptimal survival rates^5^. Consequently, enhancing the survival prospects of individuals afflicted with TSCC remains a critical imperative.

The TNM staging system, endorsed by the American Joint Committee on Cancer (AJCC), stands as a reliable and objective framework for prognostication in TSCC patients^6^. However, its efficacy in predicting post-surgical prognosis is limited, as it does not encompass lymph node heterogeneity information. Notably, several scholarly contributions propose the utility of lymph node-based parameters such as the count of favorable lymph nodes (NPLNs), ratio of favorable lymph nodes (pLNR), and logarithm of odds of favorable lymph nodes (LODDS) in prognosticating TSCC outcomes^7^-^9^. Nonetheless, the comparative prognostic accuracy of these lymph node categorization methodologies vis-à-vis the AJCC 7th TNM stage remains uncertain in TSCC cases.

Nomograms serve as valuable and user-friendly predictive tools amalgamating diverse prognostic factors to estimate patient outcomes^10^. Currently, researchers have developed numerous nomograms for precise prediction of disease progression across various malignancies, encompassing prostate cancers^11^, lung cancers 12, and colon cancers. In this study, our objective was to establish an innovative and comprehensive nomogram to predict both overall survival (OS) and cancer-specific survival (CSS) in TSCC patients undergoing surgery^13^. The provision of personalized and accurate prognostic insights through competing risk nomograms holds significant promise for guiding clinical decision-making.

## Material and Methods

### Data collection

SEER*Stat (version 8.3.9.2) was employed to access data from the Surveillance, Epidemiology, and End Results (SEER) database, a comprehensive repository covering approximately one-third of the United States population. For statistical analysis, we utilized the 'Incidence SEER 18 Registries Custom Data (with additional treatment fields), and the Nov 2020 Sub' dataset. The username utilized to retrieve the dataset was 11363-Nov2020.

The retrospective analysis and review were conducted on a study cohort comprising patients diagnosed with tonsillar squamous cell carcinoma (TSCC) as their sole primary cancer between 2010 and 2015, identified from the SEER database. A total of 1,775 patients met the inclusion criteria. Eligibility criteria included: (I) A classification of cases as "Tonsils" based on the TNM 7/CS + v0204+ schema in SEER; (II) Inclusion of TSCC patients with a diagnosis of T_1-4_N_1-3_M_0_ between 2010 and 2015, with well-documented clinical and pathological characteristics; (III) The histological type was confirmed as squamous cell carcinoma(SCC) based on positive pathology according to the International Classification of Diseases for Oncology, Third Edition (ICD-O-3); and (IV) Inclusion of patients who underwent surgical treatment. Exclusion criteria comprised: (I) Patients with incomplete or unknown clinical data; (II) Patients with a survival rate of 0 months; (III) Patients who did not undergo regional lymph node examination or lymphadenectomy; (IV) Patients with preoperative radiotherapy or multi-primary cancers; (V) Patients with incomplete information on NDLN, NPLN, the stage of the TNM, and survival outcome; (VI) Patients with tumor staging inconsistent with T_1-4_N_1-3_M_0_ according to the American Joint Committee on Cancer (AJCC) criteria. Our study primarily focused on OS and CSS. OS was defined as the interval between the onset of mortality and death from any cause, while CSS was defined as the duration until death attributed to cancer. SEER's database is regularly updated to reflect progress and prognosis, ensuring data authenticity and integrity.

Due to the limited availability of tonsil cancer samples, we expanded our validation scope to include Head and Neck Squamous Cell Carcinoma (HNSCC). Clinical data from the TCGA-HNSCC validation set were obtained from https://xenabrowser.net/datapages/ and https://gdcV18.xenahubs.net. We extracted and curated the following variables: 'age_at_index.demographic','race.demographic','gender.demographic','vital_status.demographic','ajcc_clinical_stage.diagnoses','ajcc_clinical_n.diagnoses','ajcc_clinical_t.diagnoses','treatment_type.treatments.diagnoses','initial_weight.samples','number_of_lymphnodes_positive_by_he','lymphnodes'. The exclusion criteria is equal to the SEER data screening. Additionally, we treated Disease-Specific Survival (DSS) as Cancer-Specific Survival (CSS) for subsequent survival analyses.

### Data Processing

After data filtration, the dataset underwent further categorization. Following a 7:3 ratio, patients were partitioned training and validation set. Methods such as the log odds of positive lymph nodes (LODDS) and the positive lymph node ratio (pLNR) were employed for nodal evaluation, both incorporating the count of positive lymph nodes (NPLNs). LODDS calculation followed the formula LODDS = log (NPLN + 0.50/NDLN - NPLN + 0.50), where NDLN represents the lymph nodes in total. Likewise, the pLNR = NPLN / NDLN^14^. Grades of tumor differentiation were categorized as highly differentiated (I), moderately differentiated (II), poorly differentiated (III), and undifferentiated (IV). Additionally, marital status categories encompassed married, single, widowed, and other statuses. Continuous variables relevant to survival data, such as age, NPLN, LODDS, pLNR, and tumor size, were identified using X-tile software, which determines optimal cutoff values by analyzing statistical significance^15^. The age distribution for the OS and CSS groups was divided into three categories: 29-46, 47-67, and ≥68 years for OS and 29-55, 56-61, and ≥62 years for CSS. Furthermore, the OS cohort was categorized based on LODDS as follows: -1.93 to -1.13, -1.13 to -0.71, and -0.70 to 1.66; the CSS cohort was categorized as follows: -1.93 to -1.13, -1.13 to -0.71, and -0.70 to 1.66. Furthermore, the OS and CSS groups were classified according to NPLN into the following categories: 0-1, 2-4, and ≥5. Moreover, the OS group was formed according to pLNR classifications as 0-0.05, 0.05-0.11, and 0.11-1; whereas the CSS group was formed as 0-0.06, 0.06-0.15, and 0.15-1. Additionally, tumor size categories for both OS and CSS were defined as 1-18, 19-39, and 40-150. Other variables were treated as categorical variables.

### Development of a Prognostic Model

To refine prediction models, continuous variables were discretized and expressed as ordered or discrete variables, presented in terms of frequency and percentage. Evaluation of prognostic and predictive factors' variations involved the use of the Kaplan-Meier technique and log-rank analysis. An analysis of univariate and multivariate data was conducted, with the former aimed at identifying potential prognostic factors within the training group. Data for multivariate Cox regression analysis are presented as 95% confidence intervals and hazard ratios, as well as significant predictors. The relationship between the models based on NPLN, pLNR, and LODDS was examined across the overall status, training set, and validation set. Seven distinct multivariate Cox regression models were constructed: NPLN (Model 1), pLNR (Model 2), LODDS (Model 3), pLNR + NPLN (Model 4), LODDS + NPLN (Model 5), pLNR + LODDS (Model 6), and pLNR + LODDS + NPLN (Model 7). Statistical model fitting, discriminatory ability, and accuracy were utilized to assess the predictive performance of these models.

### Construction of Nomograms

Nomograms were constructed by incorporating prognostic factors identified as independent predictors in the multivariate analysis of OS and CSS. Calibration curves were employed to juxtapose actual survival outcomes against those predicted by the nomograms, ensuring calibration across 3-, 5-, and 8-year OS and CSS timelines. In order to facilitate clinical decision-making, the decision curve analysis (DCA) was conducted to compare TNM stage efficacy with nomogram efficacy. The Akaike information criterion (AIC) was utilized to assess the adequacy of statistical model fitting, while discriminatory ability and accuracy were evaluated using Harrell's C-index^16^. Discrimination performance was further evaluated through the net reclassification index (NRI) and integrated discrimination improvement index (IDI)^17^.

## Results

### Baseline Characteristics

A cohort of 32,328 individuals diagnosed with tonsillar squamous cell carcinoma (TSCC) between January 2010 and December 2015 was initially identified for inclusion in this study. Ultimately, our study comprised a total of 1,775 patients, with 1,243 patients allocated to the training cohort and 532 patients to the validation cohort. The median age of both cohorts at diagnosis was 57 years. Both cohorts' demographic and clinical characteristics are summarized in Table 1. The majority of patients in both groups were of Black ethnicity, with 1,139 and 491 patients, respectively. Similarly, males constituted the predominant gender, with 1,023 and 446 patients, respectively. Bilateral tonsillar SCC occurrences were rare, with only five patients presenting with bilateral cancer. Notably, a significant proportion of individuals in both cohorts exhibited poorly differentiated Grade III tumors, indicative of the aggressive nature of TSCC.

### Survival Analysis

**Figure 1:**
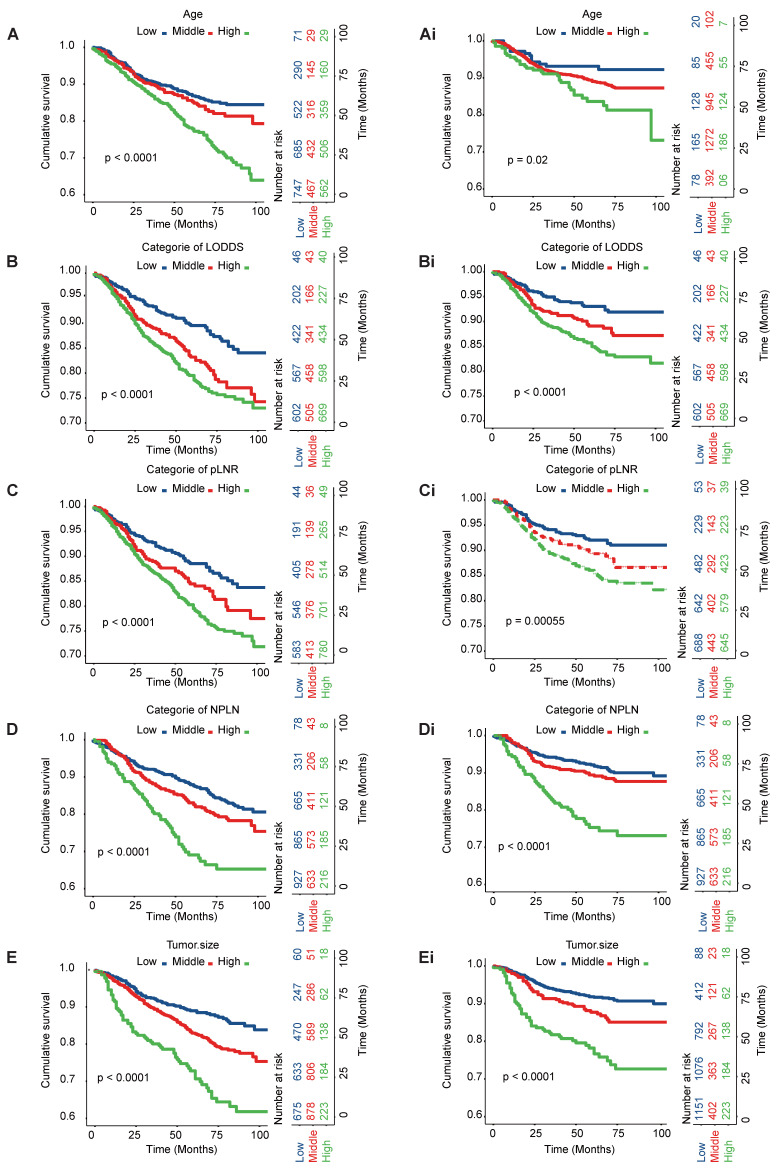
Kaplan-Meier curves of CSS and OS in high, middle and young patients [(A), OS; (Ai), CSS]; for patients with high, middle, and low LODDS [(B), OS; (Bi), CSS]; for patients with high, middle, and low pLNR [(C), OS; (Ci), CSS]; for patients with high, middle, and low NPLN [(D), OS; (Di), CSS]; for patients with high, middle, and low tumor size [(E), OS; (Ei), CSS].

**Figure 2:**
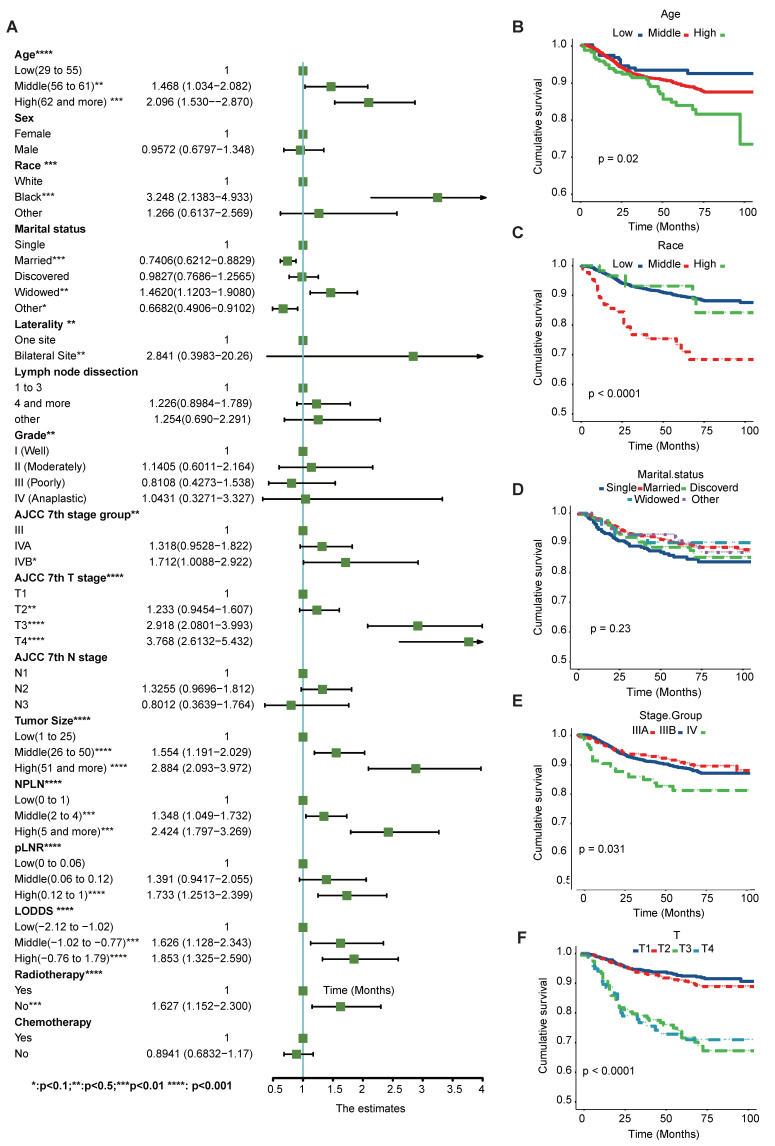
Univariate Cox regression and forest plot (A) of potential prognostic predictors for CSS. Kaplan-Meier survival curves of subgroups with significant differences (B) Age, (C) Race, (D) Marital Status, (E)Stage group, (F) T classification.

Kaplan-Meier curve analyses were conducted to ascertain both CSS and OS rates among patients diagnosed with tonsillar squamous cell carcinoma (TSCC) within the entire database (Figure 1). Notably, a larger tumor size, higher counts of NPLN, pLNR, and LODDS were significantly associated with decreased OS and CSS rates (log-rank test, p < 0.05). Figure 2 illustrates the results of univariate Cox regression analysis, demonstrating significant associations between CSS and various factors, including, race, age, tumor grade, stage group, T classification, tumor size, pLNR, LODDS, NPLN, radiotherapy, and chemotherapy (p < 0.1, Table S1).

**Figure 3:**
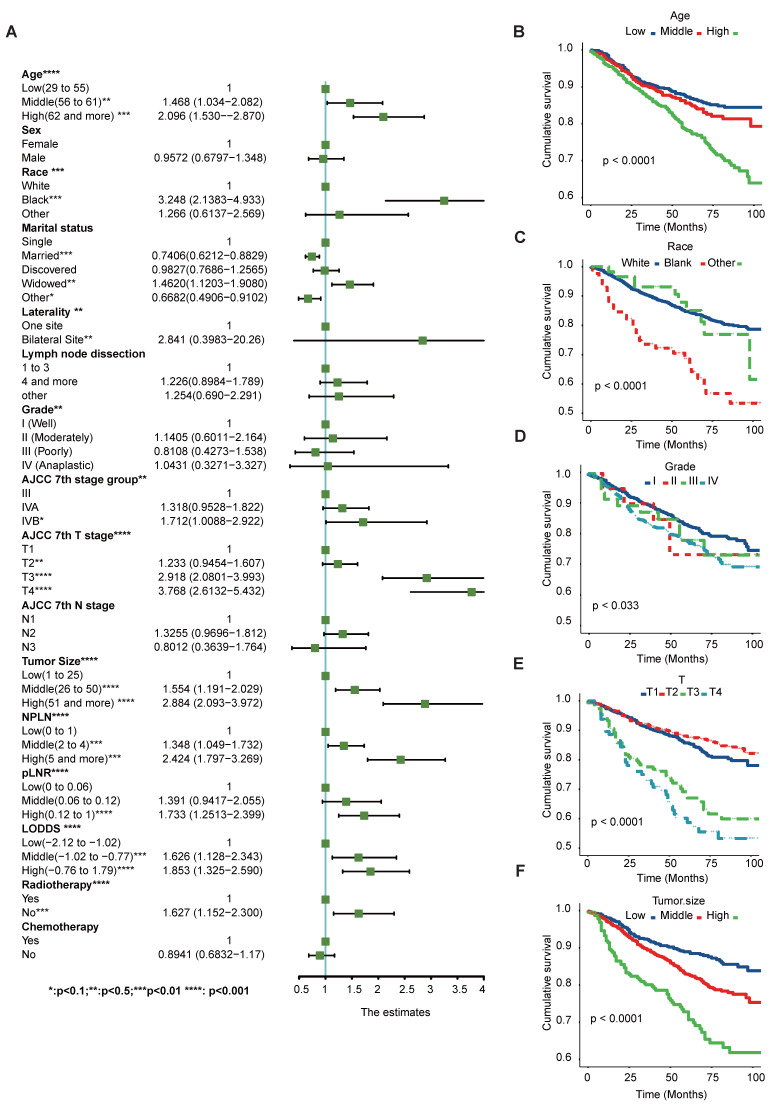
Univariate Cox regression and forest plot (A) of potential prognostic predictors for OS. Kaplan-Meier survival curves of subgroups with significant differences. (B) Age, (C) Race, (D) Grade, (E) T classification, (F) Tumor size. *Means:* p* <0.1; ** Means:* p* <0.05; ***Means: *p* <0.01.

Additionally, these results are further supported by the Kaplan-Meier and log-rank tests (p< 0.05) shown in Figure 2 and Supplementary Figure 1. Notably, chemotherapy and marital status did not exhibit significant differences in the OS cohort, as revealed by the univariate Cox regression analysis. The potential prognostic factors for OS are summarized in Figure 3, Supplementary Figure 2 and detailed in Table S2.

**Figure 4:**
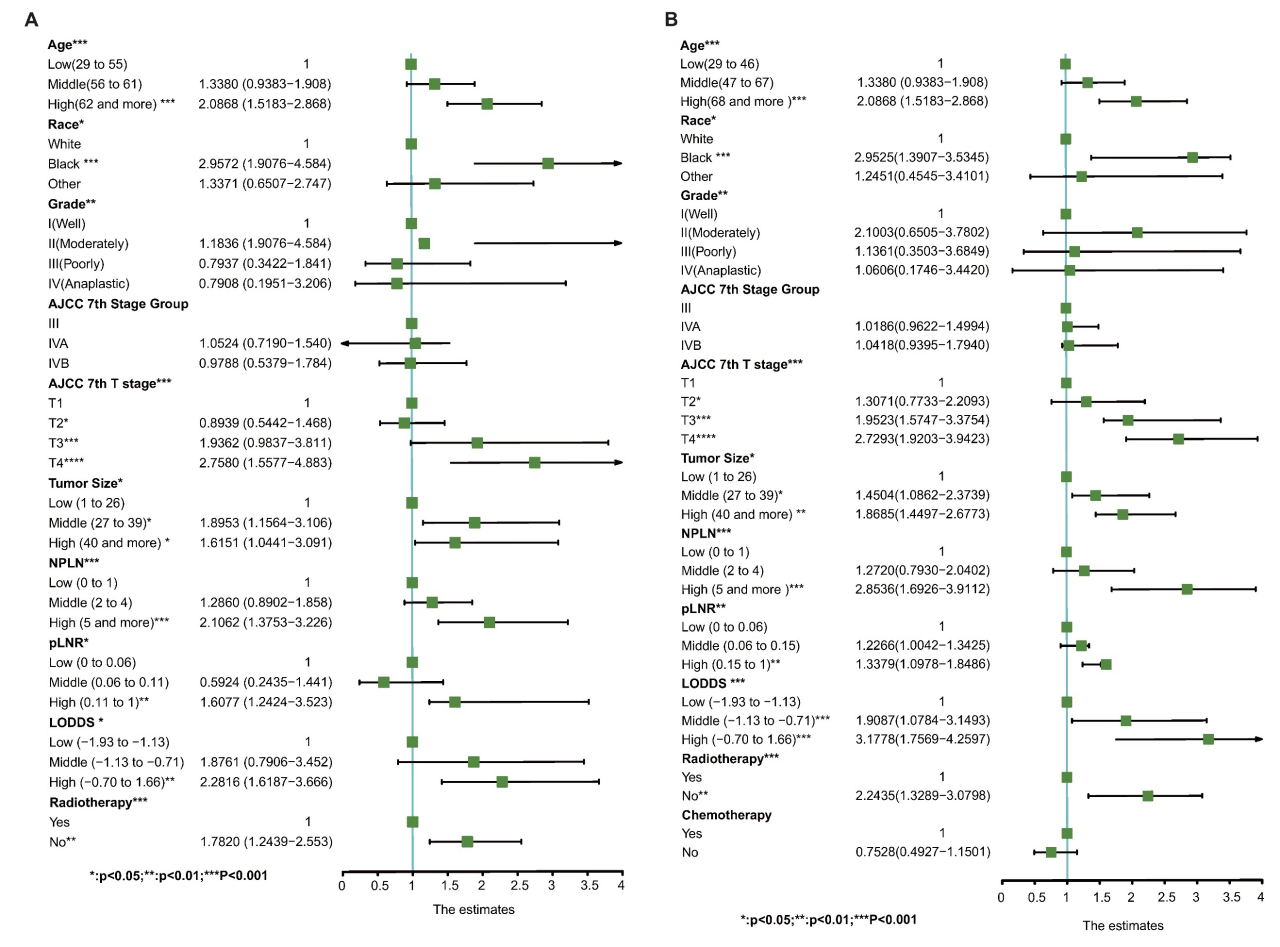
Potential prognostic predictors of CSS by multivariate cox regression and forest plots of CSS (A) and OS (B). *Means:* p* <0.1; ** Means:* p* <0.05; ***Means: *p* <0.01.

Moreover, Figure 4 delineates the results of the multivariate Cox regression model, indicating that age, race, tumor grade, stage group, T classification, tumor size, NPLN, pLNR, LODDS, and radiotherapy emerged as independent prognostic factors for CSS. Similarly, Figure 4 also identifies the variables acting as independent factors affecting prognosis for OS.

### Comparison of Prediction Performance among Models

The ranking of models was based on Akaike Information Criterion (AIC) values, serving as an indicator of model quality, where lower AIC values denote superior performance. As presented in Table 2, Model 7 demonstrated enhanced predictive accuracy, characterized by a lower AIC value compared to the other six models. In other words, Model 7 exhibited superior predictive capability. A further assessment of Model 7's predictive discriminatory ability is provided in Table 2 by incorporating Integrated Discrimination Improvement (IDI) and Net Reclassification Improvement (NRI). Neither IDI nor NRI had a p-value greater than 0.05, signifying that Model 7 displayed superior prediction performance.

### Construction of Nomogram

**Figure 5:**
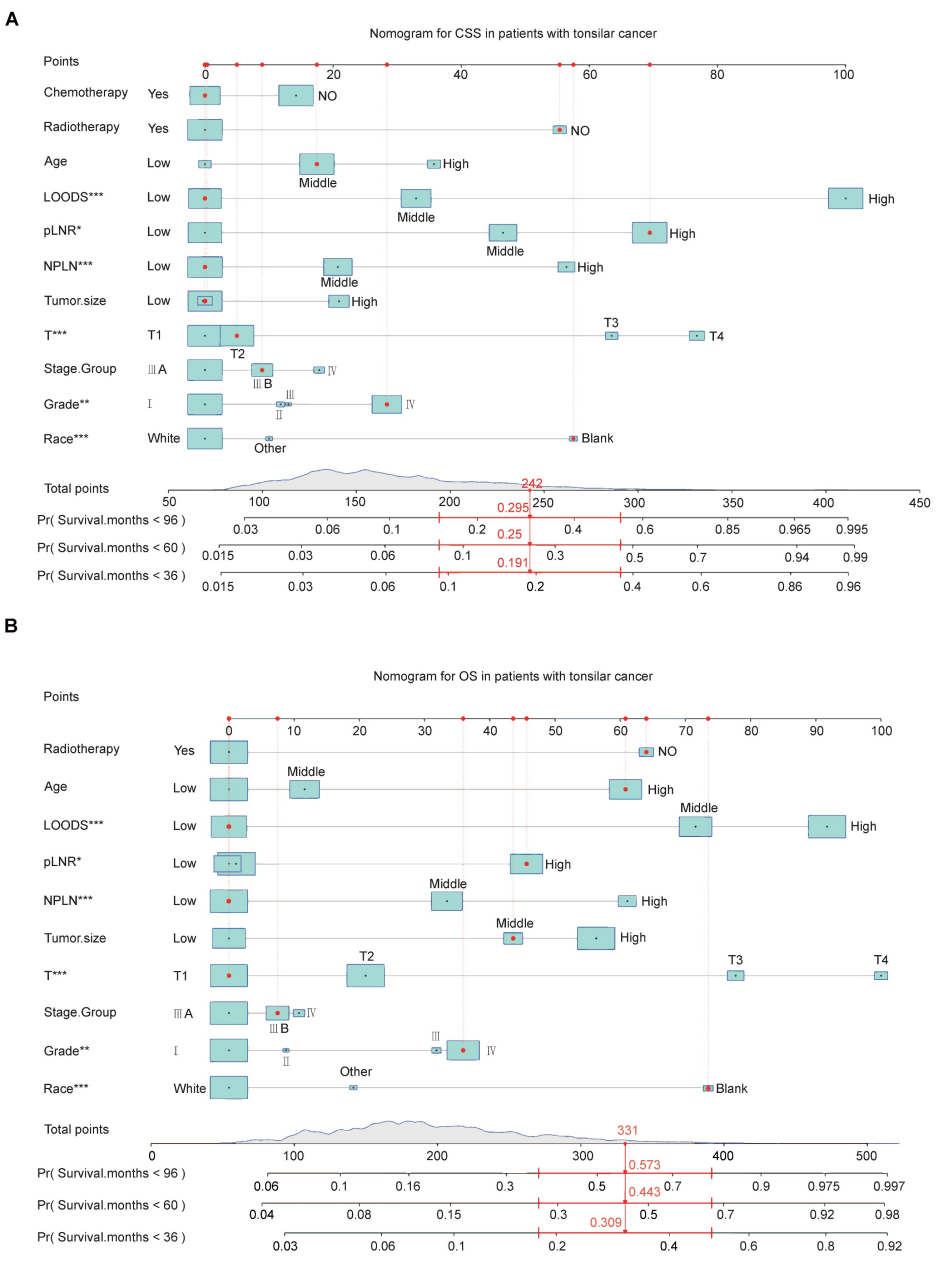
Nomograms to predict 3-, 5- and 8-year CSS (A) and OS (B) for patients.

In the training cohort, following clinical significance and statistical significance analyses for the cancer-specific survival (CSS) cohort, 11 variables were selected for inclusion in the final model after univariate and multivariate Cox regression analyses. As depicted in Figure 5, these variables encompassed age, race, tumor grade, stage group, T classification, tumor size, NPLN, pLNR, LODDS, radiotherapy, and chemotherapy. The estimation of 3-, 5-, and 8-year OS and CSS for patients with tonsillar squamous cell carcinoma (TSCC) who underwent surgery was predicated on a comprehensive calculation of a weighted score incorporating these variables. LODDS emerged as the primary factor associated with the CSS nomogram, followed by T classification, pLNR, and NPLN. Conversely, Figure 5 illustrates the nomogram representing OS, where the T classification exerted a greater influence on prognosis compared to LODDS, although LODDS remained significant in OS nomogram development. The overall score was estimated by summing the points assigned to each variable on the scoring sheet. An indication of the result was provided by a line beneath the overall score.

### Validation of Nomogram

**Figure 6:**
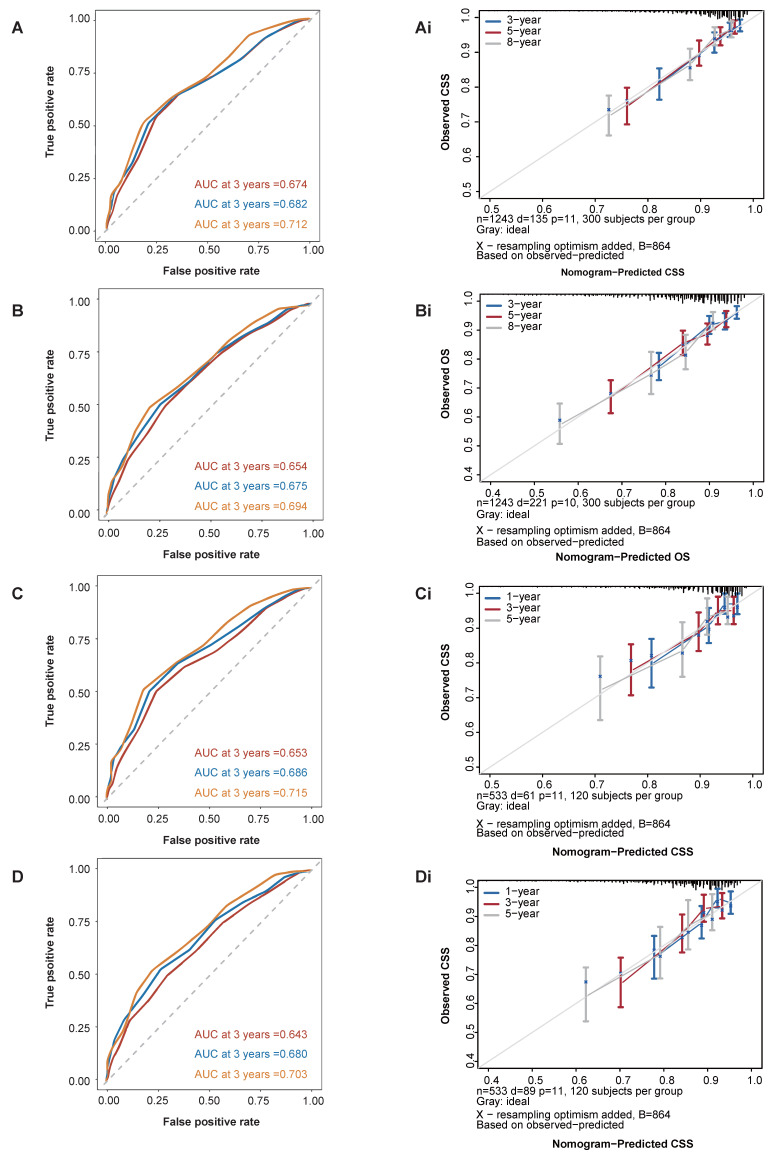
Area under curve for CSS, OS prediction of the training cohort (A, B), and external validation cohort (C, D); and calibration plots of the CSS, OS prediction of the training cohort (Ai, Bi), and external validation cohort (Ci, Di).

The CSS and OS C-indices in the training cohort were calculated using Bootstrap self-sampling, yielding values of 0.736 and 0.701, respectively (Table 2). Furthermore, the nomogram underwent both external and internal validation. The CSS and OS calibration curves for the training set (Figure 6Ai, 6Bi) and the validation set (Figure 6Ci, 6Di) closely aligned with the 45-degree line, suggesting a strong correlation between the projected nomogram and the observed survival rates over 3-, 5-, and 8-year periods. Additionally, for both the CSS and OS cohorts (Figure 6A, 6B, 6C, 6D), the nomogram demonstrated higher area under the curve (AUC) values at 3-, 5-, and 8-year intervals compared to the TNM staging system. The CSS and OS curves (Figure 6) indicated that Model 7 outperformed the AJCC 7th TNM staging system, exhibiting superior predictive ability in both training and validation set, for patient prognosis^18^.

### Risk Stratification

**Figure 7:**
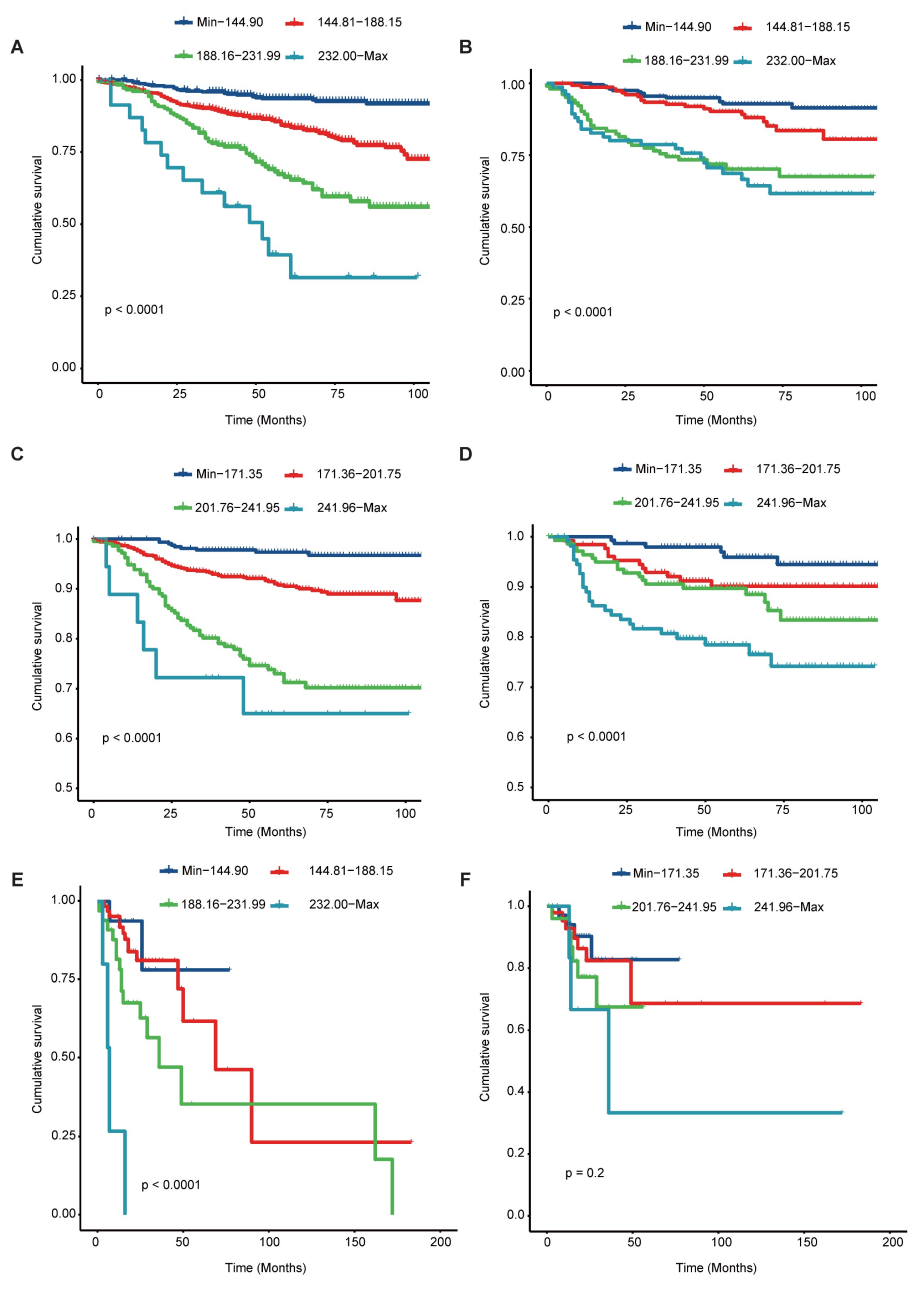
Kaplan-Meier curves of Training and Validation cohort from SEER and TCGA validation Cohort. Kaplan-Meier curves of OS from Training and Validation cohort from SEER and Validation cohort from TCGA Cohort (A, B, E); Kaplan-Meier curves of SCC from Training and Validation cohort from SEER and Validation cohort from TCGA Cohort (C, D, F).

**Figure 8:**
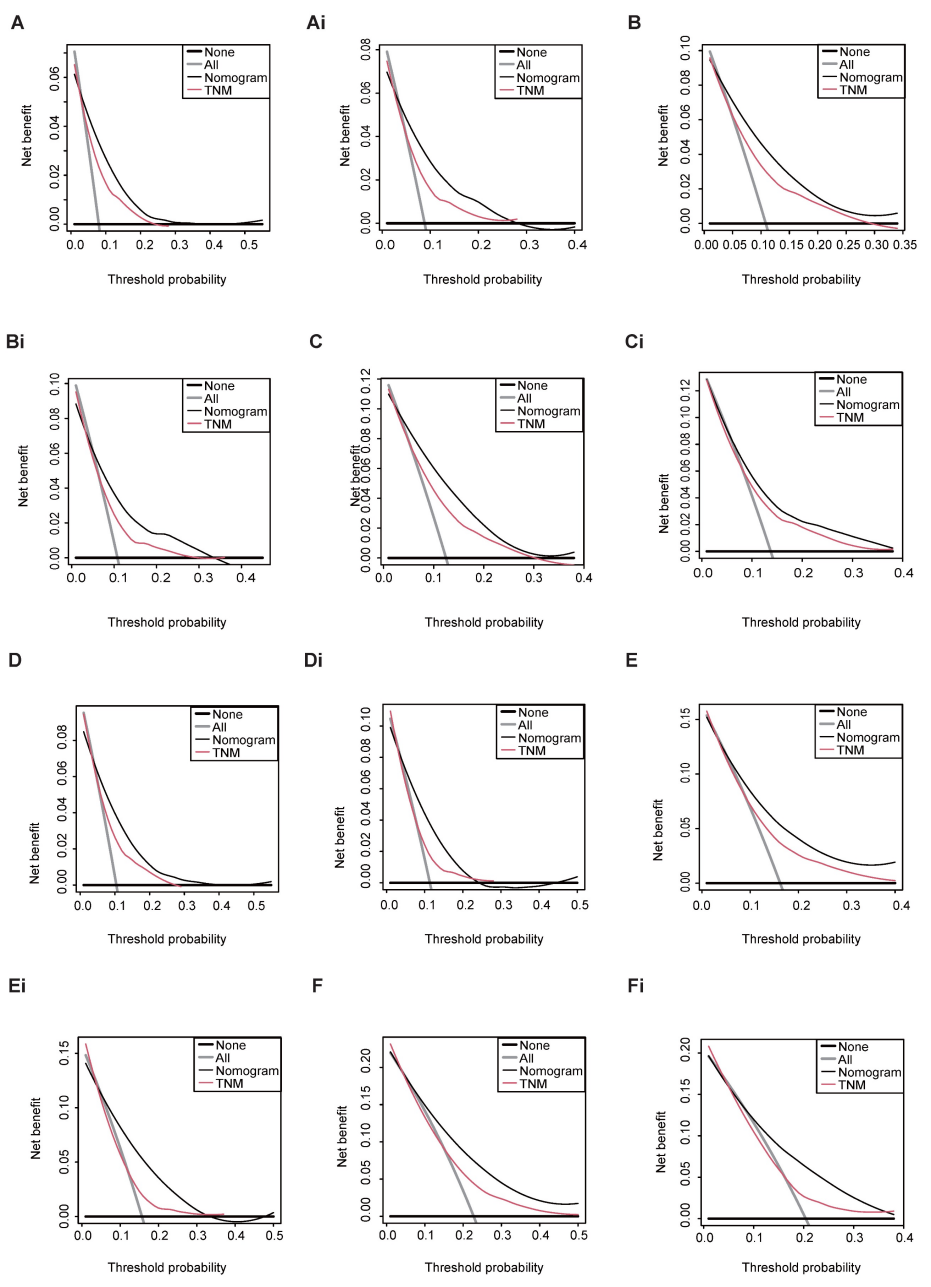
DCA of TNM stage and nomogram for 3-, 5-, and 8-year CSS (A, B, C), OS (D, E, F) prediction of the training cohort and CSS (Ai, Bi, Ci), OS (Di, Ei, Fi) prediction of the external validation cohort.

Both in the training and validation set, the scores assigned to all patients were calculated and stratified into quartiles for overall survival (OS) (ranging from minimum to 144.90, 144.81 to 188.15, 188.16 to 231.99, and 232.00 to maximum) and cancer-specific survival (CSS) (ranging from minimum to 171.35, 171.36 to 201.75, 201.76 to 241.95, and 241.96 to maximum). Notable differences were observed in CSS (Figure 7A, 7C) and OS (Figure 7B, 7D) outcomes after stratifying patients based on quartiles. Besides, we also observe the notable difference in OS (Figure 7E) from TCGA cohort. Though, the non-significant result were shown in the CSS (Figure 7F) from TCGA, which may be caused by the limited sample size. Notably, our validation through Decision Curve Analysis (DCA) demonstrated that the nomogram exhibited superior decision-making ability compared to TNM staging at 3-, 5-, and 8-year intervals (Figure 8).

## Discussion

Previous studies have indicated the beneficial impact of lymph node involvement on patient survival, emphasizing the potential predictive value of lymph nodes. The traditional AJCC staging system primarily focuses on the lymph node location in anatomy, neglecting to take the quantity and pLNRs into account. Over the past two decades, the LODDS has garnered attention following the recognition of its prognostic significance as a key element in tumor staging. Notably, in a prior investigation, Persiain et al. found LODDS to possess significant prognostic value for patient survival compared to alternative lymph node staging systems^19^. Despite this, studies elucidating its prognostic significance remain limited. LODDS, along with pLNR and NPLN, have demonstrated independent prognostic value, as endorsed by the AJCC 7th guidelines, significantly influencing prognosis. Consequently, various models were developed in this study to identify optimal prognostic indicators.

We aimed to develop a robust prognostic model for predicting outcomes in patients diagnosed with T1-4N1-3M0 TSCC following surgical intervention. Our investigation incorporated the impact of pLNR, NPLN, and LODDS on both OS and CSS. Leveraging data from the SEER database, our findings were presented visually, and the model's predictive capability was assessed using the C-index, IDI, and NRI. Additionally, DCA and calibration, graphs were generated to validate the precision and predictive power of the nomogram we developed. The collective outcomes underscore the reliability and utility of the nomograms we constructed.

Our study revealed that Model 7 exhibited superior prognostic performance compared to the conventional AJCC 7th TNM stage. This conclusion stemmed from comprehensive discussions that informed the development of both univariate and multivariate Cox regression models to predict survivorship outcomes following TSCC diagnosis. Notably, independent predictive factors such as pLNR, LODDS, and NPLN were identified and integrated into the predictive models. Moreover, the impact of radiotherapy on prognosis was elucidated, highlighting its variable effects across different head and neck cancer contexts^20^. To comprehensively assess predictive capacity, seven distinct models were devised, with LODDS emerging as a primary influencing factor, consistent with its significant role in prognostic studies across various cancer types: cervical cancer^21^, gallbladder cancer^22^, and medullary thyroid carcinoma^23^. The performance of the models we built was assessed using C-index, AIC, IDI, and NRI, all of which indicated that model 7 exhibited superior predictive capabilities. Given the competitive risk posed by different causes of mortality, Total CSS was evaluated through competing risk analysis.

Based on Model 7, we established two compelling model graphs. Internal validation was conducted using bootstrap technology, while external validation employed calibration graphs to mitigate potential biases. Well-calibrated curves observed in predicting CSS and OS underscored the reliability of our nomograms. Physicians can utilize these nomograms to assess mortality risk, guide patient counseling, and make informed treatment decisions by considering readily accessible prognostic factors. Specifically, individuals identified as having a low likelihood of survival may warrant more aggressive therapeutic interventions, such as radiotherapy.

Through a systematic evaluation of existing literature, the Gartagani, Zoi et al conducted retrospective analyses across multiple cohorts using diverse data sources, demonstrating robust evidence-based medical relevance^24^. However, its scope remained confined to analyzing the correlation between lymph node ratio (LNR) and patient survival, primarily emphasizing LNR's significance in oral cancer. While nodal quantification constitutes a critical component of TNM staging system, our model innovatively integrates multiple parameters including pathological LNR (pLNR), log odds of positive lymph nodes (LODDS), and clinical staging, enabling comprehensive prognostic evaluation. Comparative analyses revealed superior discriminative power of our model over conventional TNM staging (Figure 3D), suggesting enhanced clinical utility in tonsil cancer management.

Regarding to the research of Lee, Hojun et al, they did propose a revised N-classification system with improved C-index, but its analytical framework remains narrowly focused on nodal staging parameters^25^. The investigation described by Finegersh, Andrey et al specifically addresses recurrence patterns in clinically node-negative (cN0) patients undergoing initial surgery, which diverges from our study population^26^. Similarly, Zhang Finegersh, Andrey et al established significant correlations between lymph node metastasis incidence and T-stage/pathological staging while emphasizing prognostic impacts of TNM parameters, yet shared the methodological limitation of single-dimensional analysis observed by Gartagani, Zoi et al^24^, ^27^.

In a word, our predictive model distinctively synthesizes pLNR, LODDS, and stage group parameters through multivariable analytical approaches. This integrative strategy not only demonstrates enhanced reliability compared to traditional TNM staging but also exhibits clinical innovation through multidimensional risk stratification. The methodological advancement is further supported by rigorous validation procedures and head-to-head performance comparisons documented in our results section.

Despite the robust precision of our nomograms, certain limitations should be acknowledged. Notably, the SEER database lacks certain potential prognostic factors, including smoking history, tyrosine kinase inhibitor therapy, specific chemotherapy regimens, and immune checkpoint inhibitor therapy. Moreover, the absence of a standardized counting approach for lymph nodes may introduce inaccuracies, potentially underestimating or overestimating lymph node counts. Additionally, the SEER database predominantly represents U.S. patients, limiting its generalizability to other geographical regions.

## Conclusion

Our research highlights the improved prognostic accuracy achieved by incorporating pLNR, LODDS and NPLN in the prediction of survival outcomes for patients undergoing laryngeal surgery. Leveraging a population-based cohort, we developed a suite of nomograms tailored to forecast the 3-, 5-, and 8-year overall survival (OS) and cancer-specific survival (CSS) rates for individuals afflicted with tonsillar squamous cell carcinoma (TSCC). The utilization of these nomograms stands to empower physicians in delivering personalized and well-informed care to patients grappling with tonsil cancer, thus enhancing clinical decision-making and patient outcomes.

## Supplementary Material

Supplementary figures and tables.

## Figures and Tables

**Table 1:** Baseline characteristics of training cohort and external validation cohort.

		Total		Training cohort			External validation cohort			*p*-value
				N	%		N	%		
**Numbers of patients**		1775		1243	70.03		532	29.97		
**Age(years)**										
	Median (IQR)			57 (51, 63)			57 (51, 64)			0.5883
**Sex**										
	Female	306		220	71.90		86	28.10		0.4745
	Male	1469		1023	69.64		446	30.36		
**Race**										
	Black	1630		1139	69.88		491	30.12		1
	White	86		62	72.09		24	27.91		
	Other	59		42	71.19		17	28.81		
**Year of diagnosis**										
	2010	256		180	70.31		76	29.69		0.3599
	2011	308		232	75.32		76	24.68		
	2012	270		183	67.78		87	32.22		
	2013	305		208	68.20		97	31.80		
	2014	331		229	69.18		102	30.82		
	2015	305		211	69.18		94	30.82		
**Laterality**										
	One site	1771		1241	70.07		530	29.93		0.7921
	Bilateral site	4		2	50.00		2	50.00		
**Marital status**										
	Single	296		225	76.01		71	23.99		0.09514
	Married	1081		753	69.66		328	30.34		
	Discovered	201		133	66.17		68	33.83		
	Widowed	42		30	71.43		12	28.57		
	Other	155		102	65.81		53	34.19		
**Grade**										
	I (Well)	57		40	70.18		17	29.82		0.09251
	II (Moderately)	719		527	73.30		192	26.70		
	III (Poorly)	975		659	67.59		316	32.41		
	IV (Anaplastic)	24		17	70.83		7	29.17		
Lymph node dissection										
	1 to 3	240		168	70.00		72	30.00		0.968
	4 and more	1450		1015	70.00		435	30.00		
	other	85		60	70.59		25	29.41		
**AJCC 7th stage group**										
	III	451		329	72.95		122	27.05		0.2476
	IVA	1212		834	68.81		378	31.19		
	IVB	112		80	71.43		32	28.57		
**AJCC 7th T stage**										
	T1	775		533	68.77		242	31.23		0.306
	T2	755		531	70.33		224	29.67		
	T3	149		104	69.80		45	30.20		
	T4	96		75	78.13		21	21.88		
**AJCC 7th N stage**										
	N1	468		343	73.29		125	26.71		0.1978
	N2	1225		843	68.82		382	31.18		
	N3	82		57	69.51		25	30.49		
**Radiotherapy**										
	Yes	1550		1086	70.06		464	29.94		0.9921
	NO	225		157	69.78		68	30.22		
**Chemotherapy**										
	Yes	1004		694	69.12		310	30.88		0.3697
	No	771		549	71.21		222	28.79		
**NDLN**										
	Median (IQR)			24 (10, 38.5)				24 (11,38)		0.479
**NPLN**										
	Median (IQR)			1 (1, 3)				1 (1, 3)		0.9655
**Tumor Size(mm)**										
	Median (IQR)			22 (15,30)				26 (18-40)		0.4646
**pLNR**										
	Median (IQR)			0.091 (0.04, 0.279)				0.087 (0.037, 0.025)		0.2839
**LODDS**										
	Median (IQR)			-0.914 ( -1.929, -0.368)				-0.934 (-1.263, -0.444)		0.1947
										

**Table 2:** Comparison of prediction performance among models

Model	AIC	NRI (95%CI)	*p* value	IDI (95% CI)	*p* value
Cancer-specific survival					
Model 1	2,762.49	-0.018 (-0.116 to -0.009)	0.002	-0.008 (-0.033 to -0.001)	< 0.001
Model 2	2,767.97	-0.02 (-0.139 to 0.004)	0.063	-0.016 (-0.033 to -0.004)	< 0.001
Model 3	2,762.52	-0.023 (-0.126 to -0.001)	0.0019	-0.012 (-0.0331 to -0.003)	< 0.001
Model 4	2,759.82	-0.007 (-0.100 to -0.002)	0.021	-0.007 (-0.022 to -0.001)	< 0.001
Model 5	2,755.14	-0.05 (-0.09 to -0.004)	0.016	-0.004 (-0.017 to 0.000)	0.033
Model 6	2,763.10	-0.006 (-0.119-0.039)	0.075	-0.010 (-0.027 to -0.002)	< 0.001
Model 7	2,751.52	Reference		Reference	
Overall survival					
Model 1	4,333.01	-0.051 (-0.097 to -0.032)	< 0.001	-0.007 (-0.020 to -0.001)	0.007
Model 2	4,335.80	-0.038 (-0.141 to -0.012)	0.006	-0.014 (-0.030 to -0.005)	< 0.001
Model 3	4,332.17	-0.031 (-0.128 to -0.023)	< 0.001	-0.010 (-0.024 to -0.002)	0.007
Model 4	4,328.25	-0.017 (-0.065 to 0.019)	0.0079	-0.006 (-0.018 to 0.000)	0.02
Model 5	4,323.76	-0.002 (-0.021 to 0.058)	0.15	-0.002 (-0.010 to 0.000)	0.045
Model 6	4,334.17	-0.031 (-0.109 to 0.253)	0.0088	-0.010 (-0.022 to -0.002)	0.001
Model 7	4314.98	Reference		Reference	

## Abbreviations

TSCC: Tonsillar squamous cell carcinoma
NPLNs: number of positive lymph nodes
pLNRs: ratio of positive lymph nodes
LODDS: logarithm of the odds of positive lymph nodes
SEER: Surveillance, Epidemiology, and End Results database
C-index: Harrell's concordance index
AIC: Akaike information criterion
IDI: Integrated Discrimination Improvement
NRI: Net Reclassification Improvement
SCC: squamous cell carcinoma
AJCC: American Joint Committee on Cancer
OS: overall survival
CSS: cancer-specific survival
