# Supplementary material for: Nomograms Combining Three Different Lymph Node Classifications to Predict the Survival of Tonsillar Squamous Cell Carcinoma Patients Undergoing Surgical Treatment
Source: J Cancer. 2025 Jul 28;16(12):3599–614. doi: 10.7150/jca.98658 (PMC12435307; doi:10.7150/jca.98658)
Supplement: Supplementary file 1 — Supplementary figures and tables. [file jcav16p3599s1.zip › Supplementary materials.pdf]

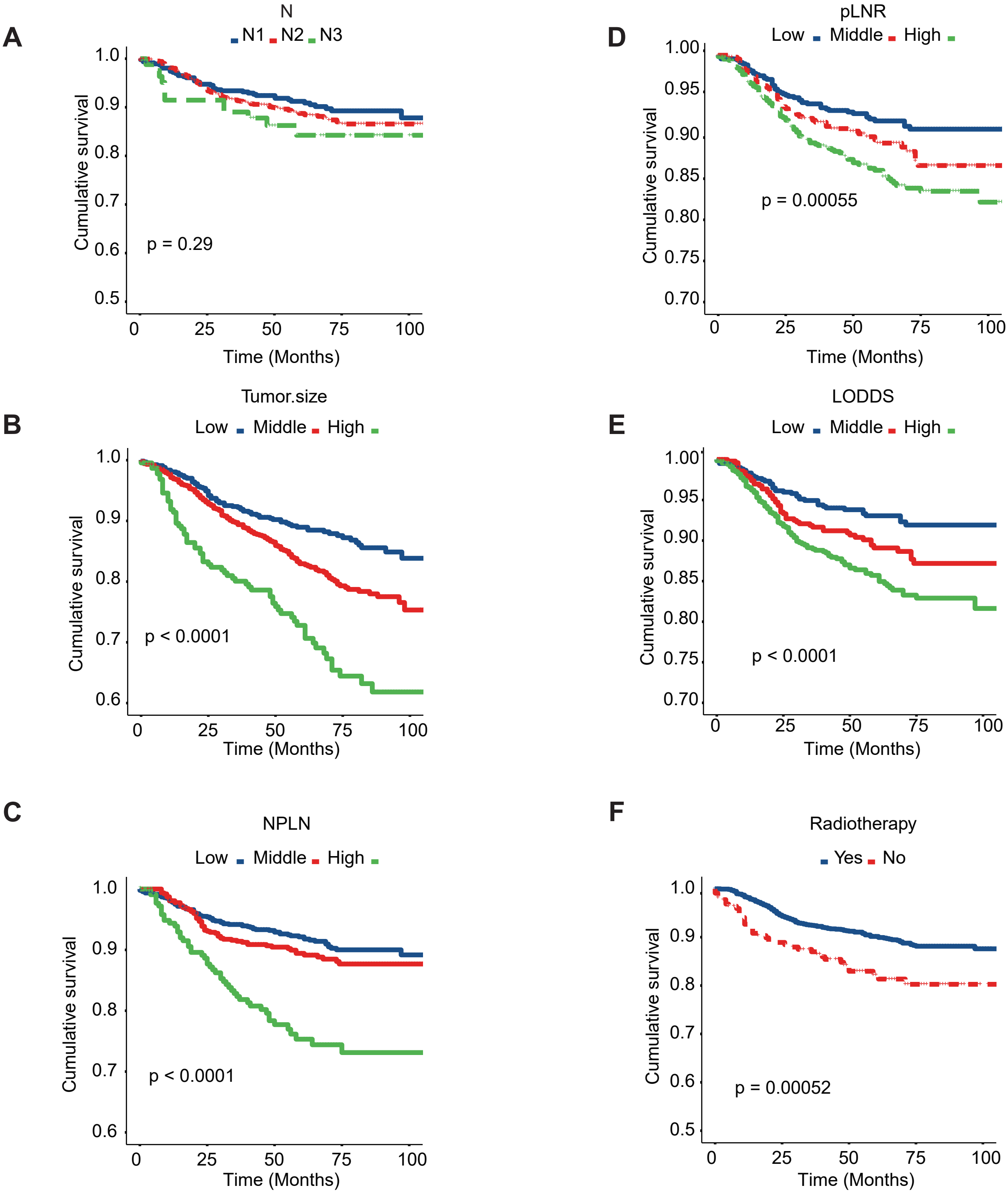
A
N
N1 N2 N3
Cumulative survival
1.0
0.9
0.8
0.7
0.6
0.5
p = 0.29
0
25
50
75
100
Time (Months)
D
pLNR
Low Middle High
Cumulative survival
1.00
0.95
0.90
0.85
0.80
0.75
0.70
p = 0.00055
Time (Months)
B
Tumor.size
Low Middle High
Cumulative survival
p < 0.0001
Time (Months)
E
LODDS
Low Middle High
Cumulative survival
p < 0.0001
Time (Months)
C
NPLN
Low Middle High
Cumulative survival
p < 0.0001
Time (Months)
F
Radiotherapy
Yes No
Cumulative survival
p = 0.00052
Time (Months)

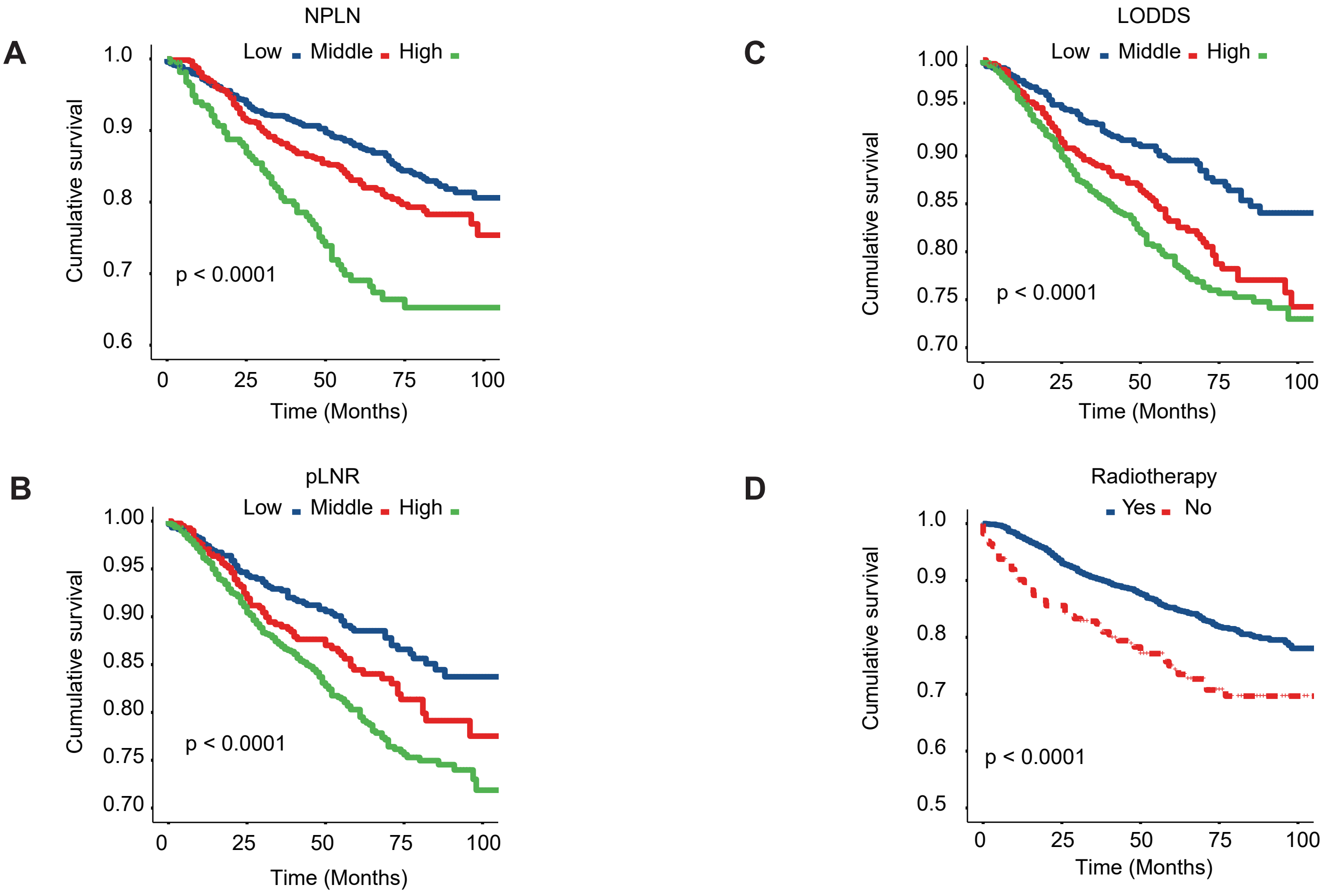
A
NPLN
Low Middle High
Cumulative survival
1.0
0.9
0.8
0.7
0.6
p < 0.0001
0
25
50
75
100
Time (Months)
C
LODDS
Low Middle High
Cumulative survival
1.00
0.95
0.90
0.85
0.80
0.75
0.70
p < 0.0001
0
25
50
75
100
Time (Months)
B
pLNR
Low Middle High
Cumulative survival
1.00
0.95
0.90
0.85
0.80
0.75
0.70
p < 0.0001
0
25
50
75
100
Time (Months)
D
Radiotherapy
Yes No
Cumulative survival
1.0
0.9
0.8
0.7
0.6
0.5
p < 0.0001
0
25
50
75
100
Time (Months)

Table S1. Univariate Cox regression and multivariate cox regression analysis of potential prognostic predictors for CSS.

Characteristics		Univariate analysis		Multivariate analysis	
		HR (95%CI)	*p* value	HR (95%CI)	*p* value
Age			0.005		0.038
	Low (29 to 46)	1 (1,1)		1 (1,1)	
	Middle (47 to 67)	1.632 (1.0269-2.221)		1.3380 (0.9383-1.908)	
	High (68 and more)	1.892 (1.0431-3.246)		2.0868 (1.5183-2.868)	
Sex			0.9		
	Female	1 (1,1)			
	Male	1.029 (0.657-1.612)			
Race			0.003		0.016
	White	1 (1,1)		1 (1,1)	
	Black	2.208 (1.6122-3.780)		2.9572 (1.9076-4.584)	
	Other	1.034 (0.3813-2.804)		1.3371 (0.6507-2.747)	
Marital status			0.135		
	Single	1 (1,1)			
	Married	0.7081 (0.46701-1.074)			
	Discovered	0.8429 (0.46099=1.541)			
	Widowed	0.2276 (0.03107-1.667)			
	Other	0.5865 (0.27921-1.232)			
Grade			0.0411		0.0063
	I (Well)	1 (1,1)		1 (1,1)	
	II (Moderately)	1.827 (0.5759-5.798)		2.1003 (0.6505-3.7802)	
	III (Poorly)	1.108 (0.3471-3.539)		1.1361 (0.3503-3.6849)	
	IV (Anaplastic)	1.660 (0.2773-9.938)		1.0606 (0.1746-3.4420)	
AJCC 7th stage group			0.0411		0.87
	III	1 (1,1)		1 (1,1)	
	IVA	1.452 (0.9297-2.184)		1.0186 (0.9622-1.4994)	
	IVB	1.902 (0.9636-3.755)		1.0418 (0.9395-1.7940)	
AJCC 7th T classification			<0.001		<0.001
	T1	1 (1,1)		1 (1,1)	
	T2	1.527 (0.9946-2.344)		1.3071 (0.7733-2.2093)	
	T3	2.672 (1.8420-3.682)		1.9523 (1.5747-3.3754)	
	T4	3.409 (2.7751-3.935)		2.7293 (1.9203-3.9423)	
AJCC 7th N stage			0.228		
	N1	1 (1,1)			
	N2	1.447 (0.9573-2.187)			
	N3	1.015 (0.3927-2.621)			
Tumor Size			<0.001		0.063
	Low (1 to 26)	1 (1,1)		1 (1,1)	
	Middle (27 to 39)	1.681 (1.125-2.511)		1.4504 (1.0862-2.3739)	
	High (40 and more)	2.984 (1.960-3.542)		1.8685 (1.4497-2.6773)	
NPLN			<0.001		<0.001
	Low (0 to 1)	1 (1,1)		1 (1,1)	
	Middle (2 to 4)	1.171 (0.7844-1.748)		1.2720 (0.7930-2.0402)	
	High (5 and more)	2.249 (1.1441-3.922)		2.8536 (1.6926-3.9112)	
pLNR			<0.001		0.034
	Low (0 to 0.06)	1 (1,1)		1 (1,1)	
	Middle (0.06 to 0.15)	1.353 (0.8455-2.166)		1.2266 (1.0042-1.3425)	
	High (0.15 to 1)	1.820 (1.2200-2.716)		1.3379 (1.0978-1.8486)	
LODDS			<0.001		<0.001
	Low (-1.93 to -1.13)	1 (1,1)		1 (1,1)	
	Middle (-1.13 to -0.71)	1.695 (1.040-2.762)		1.9087 (1.0784-3.1493)	
	High (-0.70 to 1.66)	2.233 (1.439-3.464)		3.1778 (1.7569-4.2597)	
Radiotherapy			0.0275		<0.001
	Yes	1 (1,1)		1 (1,1)	
	No	1.642 (1.056-2.553)		2.2435 (1.3289-3.0796)	
Chemotherapy			0.0974		0.15
	Yes	1 (1,1)		1 (1,1)	
	No	0.7211 (0.5069-1.026)		0.7528 (0.4927-1.1501)	

HR hazard ratio, CI confidence interval

Table S2. Univariate Cox regression and multivariate cox regression analysis of potential prognostic predictors for OS.

Characteristics		Univariate analysis		Multivariate analysis	
		HR (95%CI)	*p* value	HR (95%CI)	*p* value
Age			<0.001		<0.001
	Low (29 to 46)	1 (1,1)		1 (1,1)	
	Middle (47 to 67)	1.468 (1.034-2.082)		1.3380 (0.9383-	
	High (68 and more)	2.096 (1.530--2.870)		2.0868 (1.5183-	
Sex			0.297		
	Female	1 (1,1)			
	Male	0.9572 (0.6797-1.348)			
Race			0.0089		0.0025
	White	1 (1,1)		1 (1,1)	
	Black	3.248 (2.1383-4.933)		2.9572 (1.9076-	
	Other	1.266 (0.6137-2.569)		1.3371 (0.6507-	
Marital status			0.475		
	Single	1 (1,1)			
	Married	0.7406 (0.6212-			
	Discovered	0.9827 (0.7686-			
	Widowed	1.4620 (1.1203-			
	Other	0.6682 (0.4906-			
Grade			0.0128		0.0067
	I (Well)	1 (1,1)		1 (1,1)	
	II (Moderately)	1.1405 (0.6011-2.164)		1.1836 (1.9076-	
	III (Poorly)	0.8108 (0.4273-1.538)		0.7937 (0.3422-	
	IV (Anaplastic)	1.0431 (0.3271-3.327)		0.7908 (0.1951-	
AJCC 7th stage group			0.0358		0.82
	III	1 (1,1)		1 (1,1)	
	IVA	1.318 (0.9528-1.822)		1.0524 (0.7190-	
	IVB	1.712 (1.0088-2.922)		0.9788 (0.5379-	
AJCC 7th T classification			<0.001		<0.001
	T1	1 (1,1)		1 (1,1)	
	T2	1.233 (0.9454-1.607)		0.8939 (0.5442-	
	T3	2.918 (2.0801-4.093)		1.9362 (0.9837-	
	T4	3.768 (2.6132-5.432)		2.7580 (1.5577-	
AJCC 7th N classification			0.307		
	N1	1 (1,1)			
	N2	1.3255 (0.9696-1.812)			
	N3	0.8012 (0.3639-1.764)			
Tumor Size			<0.001		0.18
	Low (1 to 26)	1 (1,1)		1 (1,1)	
	Middle (27 to 39)	1.554 (1.191-2.029)		1.8953 (1.1564-	
	High (40 to 150)	2.884 (2.093-3.972)		1.6151 (0.8441-	
NPLN			<0.001		
	Low (0 to 1)	1 (1,1)		1 (1,1)	<0.001
	Middle (2 to 4)	1.348 (1.049-1.732)		1.2860 (0.8902-	
	High (5 to 68)	2.424 (1.797-3.269)		2.1062 (1.3753-	
pLNR			<0.001		0.25
	Low (0 to 0.06)	1 (1,1)		1 (1,1)	
	Middle (0.06 to 0.11)	1.391 (0.9417-2.055)		0.5924 (0.2435-	
	High (0.11 to 1)	1.733 (1.2513-2.399)		1.6077 (0.2424-	
LODDS			<0.001		0.045
	Low (-1.93 to -1.13)	1 (1,1)		1 (1,1)	
	Middle (-1.13 to -0.71)	1.626 (1.128-2.343)		1.8761 (0.7906-	
	High (-0.70 to 1.66)	1.853 (1.325-2.590)		2.2816 (0.9187-	
Radiotherapy			<0.001		<0.001
	Yes	1 (1,1)		1 (1,1)	
	No	1.627 (1.152-2.3)		1.7820 (1.2439-	
Chemotherapy			0.467		
	Yes	1 (1,1)			
	No	0.8941 (0.6832-1.17)			

HR hazard ratio, CI confidence interval
