# Supplementary material for: Nomograms Combining Three Different Lymph Node Classifications to Predict the Survival of Tonsillar Squamous Cell Carcinoma Patients Undergoing Surgical Treatment
Source: J Cancer. 2025 Jul 28;16(12):3599–614. doi: 10.7150/jca.98658 (PMC12435307; doi:10.7150/jca.98658)
Supplement: Supplementary file 1 — Supplementary figures and tables. [file jcav16p3599s1.zip › supplementary figure legends.pdf]

Supplementary Figure1. Kaplan-Meier survival curves of subgroups with significant differences (A) N classification, (B) Tumor size, (C) NPLN, (D) pLNR, (E) LODDS, (F) Radiotherapy.

Supplementary Figure2. Kaplan-Meier survival curves of subgroups with significant differences. (A) NPLN, (B) pLNR, (C) LODDS, (D) Radiotherapy.
